# Survey dataset of factors affecting Islamic donation intention in Malaysia

**DOI:** 10.1016/j.dib.2024.110714

**Published:** 2024-07-09

**Authors:** Mohamad Syahmi Mat Daud, Hairunnizam Wahid, Riayati Ahmad, Raudha Md. Ramli

**Affiliations:** Faculty of Economics and Management, Universiti Kebangsaan Malaysia, 43600, Malaysia

**Keywords:** Islamic donation, Theory of planned behavior, Religious commitment, Institutional trust, Malaysia

## Abstract

The present dataset investigates the factors that influence Malaysian Muslims' Islamic donation intentions. The research model was developed based on the integration of the theory of planned behavior (TPB) and social capital theory with six latent variables. A self-administrated survey of 400 Muslims with various demographic characteristics yielded the dataset in February 2024. The dataset was the basis for identifying factors that influence Muslims' Islamic donation intentions, thereby helping scholars and Islamic non-profit organisations understand how Malaysian Muslims participated in donation activities by interacting with religious commitment, institutional trust, attitude, subjective norms, and perceived behavioral control.

Specifications Table

**Table:** 

Subject	Economics, Econometrics and Finance.
Specific subject area	Behavioural Finance and Economics.
Type of data	Table.
Data collection	A survey method through a questionnaire were used to collect the primary data.
Data source location	Region: Asia Country: Malaysia.
Data accessibility	Repository name: Survey dataset of factors affecting Islamic donation intention in Malaysia Data identification number: 10.17632/b244w8sftm.1 Direct URL to data: https://data.mendeley.com/datasets/b244w8sftm/1
Related research article	None.

## 1 Value of the Data

• The data will be useful in identifying factors that influence Muslims' intentions to donate money in Malaysia especially during the COVID-19 pandemic recovery.
• The dataset will be useful to predict the effect of religious commitment and institutional trust on donations in Malaysia.
• The data is valuable to highlight important issues related to giving behavior as a practical response for vulnerable groups in Malaysia.
• Policymakers, stakeholders, the government, researchers, Muslim scholars, and non-profit organizations can use the data to target potential donors for the poor and vulnerable groups. Furthermore, the data provided insights that allowed the parties to outline the strategy and plans to increase charity.

## 2 Background

Understanding the factors affecting donation intention is crucial because it helps charity organizations tailor their strategies effectively in several ways. First, by understanding what influences people's willingness to donate, organizations can craft targeted campaigns. Secondly, the agency can allocate resources efficiently and foster stronger connections with potential donors. Ultimately, this increases the likelihood of achieving their fundraising goals. On the other hand, religion and institutions are said to have a strong influence on encouraging their followers to make donations [1,2]. Hypothetically, religious commitment can inspire individuals to donate, while collecting institutions provide assurances that the collected funds will be utilized appropriately. Together, these factors significantly influence individuals' intentions to participate in donation activities. Specifically, commitment or a sense of duty to help others that stems from religious teachings leads to higher rates of charitable giving, especially among religious individuals. This includes a moral obligation that emphasizes altruism, generosity, and compassion. While trust in charitable organizations pertains to the confidence donors have in institutions, especially religious institutions, to utilize donated funds effectively and ethically. Therefore, higher levels of institutional trust can lead to an increased willingness to donate, as donors believe their contributions will be used for meaningful purposes [2].

## 3 Data Description

The dataset was obtained through a quantitative research technique by collecting primary data using a cross-sectional survey questionnaire. The data collection process started from February 2024 until April 2024 via an online survey, as it is the most convenient approach and the only option available for some. A Google Form was used to create an online survey that was sent through online platforms such as email, Facebook, and WhatsApp. The data collection process managed to generate 600 responses. After removing all incomplete replies, a final set of 400 responses was usable for further analysis. The response rate was 66.7 %.

**Fig. 1:**
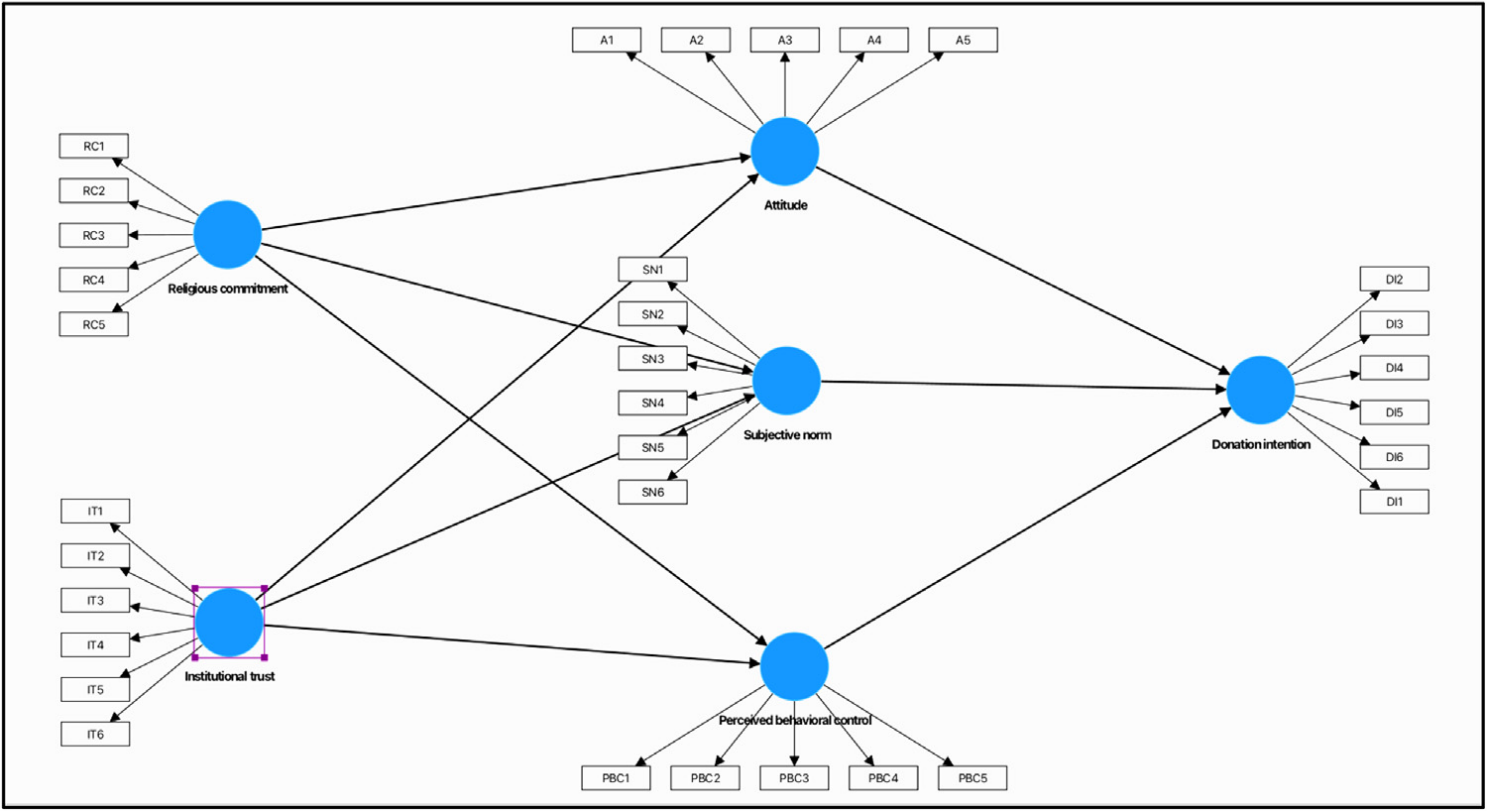
Relationship among the construct variables.

The questionnaire has four parts, where Part A aims to gather the demographic profile of the respondents. This includes gender, age, education level, household income, marital status, number of dependents, and employment. The second questionnaire consists of 11 items measuring the constructs of religious commitment and institutional trust. Part C contains 16 items measuring the variables for attitude, subjective norm, and perceived behavioral control. The final section of D is on donation intention (dependent variable), in which six items were used to measure the variable (Fig. 1).

This study selects attitude, subjective norm, and perceived behavioral control as the basis of the theory of planned behavior (TPB). Attitudes, subjective norms, and perceived behavioral control collectively influence an individual's intention, which in turn predicts actual behavior. While religious commitment and institutional trust are important in social capital theory to understand how social relationships, norms, and institutions shape individual and collective behaviors, attitudes, and outcomes in society.

According to Ajzen [3], the variables in the Theory of Planned Behavior (TPB) are defined as follows: Attitude: an individual's positive or negative feelings (evaluative judgments) toward performing a particular behavior. It reflects how favorable or unfavorable a person perceives the behavior to be; Subjective norm: the perceived social pressure or influence to perform or not perform a behavior. It includes the person's beliefs about whether important others (such as friends, family, or colleagues) think they should or should not engage in the behavior, as well as the person's motivation to comply with these perceived expectations; Perceived behavioral control: the perceived ease or difficulty of performing a behavior. Factors such as self-efficacy (confidence in one's ability to perform the behavior) and perceived constraints or barriers that may affect the ability to carry out the behavior are included.

Religious commitment can be defines as an individual's dedication, involvement, and adherence to religious beliefs, practices, and institutions. It encompasses the extent to which a person identifies with and participates in religious activities, rituals, and communities. Religious commitment can include beliefs, behaviors, and affiliations that contribute to a person's social networks and interactions within religious contexts [4]. While Institutional trust is the confidence and belief that individuals have in various institutions within society, such as the government, corporations, NGOs, and religious organizations. It reflects the expectation that these institutions will act in a trustworthy manner, uphold their responsibilities, and serve the interests of society and its members. Institutional trust is essential for fostering cooperation, collaboration, and social cohesion within communities and across different societal sectors [5].

Table 2 presents the convergent validity of the model. It shows that factor loading ranges from 0.634 to 0.887, which is above the threshold value of 0.5, suggested by Bagozzi et al. [6]. The dataset composite reliability (CR) ranges from 0.834 to 0.910 and AVE is in a range from 0.502 to 0.670, which is above the threshold values of 0.70 and 0.5 as recommended by Vatamanescu et al. [7].

**Table 1:** Description of the latent constructs.

Constructs	Items	Mean	Median	Std. deviation
Donation intention (DI)	DI1	4.200	4.000	0.648
	DI2	4.308	4.000	0.602
	DI3	4.220	4.000	0.715
	DI4	4.053	4.000	0.768
	DI5	4.220	4.000	0.605
	DI6	4.418	4.000	0.603
Religious commitment (RC)	RC1	4.312	4.000	0.682
	RC2	4.325	4.000	0.717
	RC3	4.440	5.000	0.630
	RC4	4.345	4.000	0.660
	RC5	4.285	4.000	0.761
Institutional trust (IT)	IT1	3.877	4.000	0.795
	IT2	3.830	4.000	0.831
	IT3	3.980	4.000	0.659
	IT4	4.010	4.000	0.682
	IT5	3.720	4.000	0.823
	IT6	3.973	4.000	0.649
Attitude (A)	A1	4.420	4.000	0.607
	A2	4.482	5.000	0.600
	A3	4.025	4.000	0.774
	A4	4.242	4.000	0.666
	A5	4.107	4.000	0.732
Subjective Norm (SN)	SN1	4.223	4.000	0.631
	SN2	3.842	4.000	0.799
	SN3	3.795	4.000	0.865
	SN4	3.955	4.000	0.706
	SN5	3.955	4.000	0.702
	SN6	3.913	4.000	0.731
Perceived behavioral control (PBC)	PBC1	3.877	4.000	0.792
	PBC2	4.152	4.000	0.806
	PBC3	4.090	4.000	0.683
	PBC4	4.215	4.000	0.659
	PBC5	4.005	4.000	0.781

**Table 2:** Convergent validity of the model.

Constructs	Items	Factor loadings (>0.5)	Composite reliability (>0.7)	AVE (>0.5)
Donation intention (DI)	DI1	0.770	0.862	0.512
	DI2	0.828		
	DI3	0.687		
	DI4	0.694		
	DI5	0.630		
	DI6	0.667		
Religious commitment (RC)	RC1	0.733	0.910	0.670
	RC2	0.784		
	RC3	0.876		
	RC4	0.887		
	RC5	0.804		
Institutional trust (IT)	IT1	0.796	0.906	0.617
	IT2	0.808		
	IT3	0.780		
	IT4	0.837		
	IT5	0.744		
	IT6	0.745		
Attitude (A)	A1	0.794	0.846	0.524
	A2	0.748		
	A3	0.681		
	A4	0.746		
	A5	0.641		
Subjective Norm (SN)	SN1	0.683	0.892	0.579
	SN2	0.798		
	SN3	0.741		
	SN4	0.788		
	SN5	0.794		
	SN6	0.756		
Perceived behavioral control (PBC)	PBC1	0.634	0.834	0.502
	PBC2	0.715		
	PBC3	0.682		
	PBC4	0.728		
	PBC5	0.774		

Table 3 presents the discriminant analysis based on two criteria. The first criteria showed that the square root of the average variances extracted (AVEs) was all greater than the correlations among constructs, suggesting adequate discriminant validity [8]. The second criterion of the heterotrait-monotrait ratio (HTMT) indicates that the values fall within the threshold range of 0.85 to 0.90. The results of the analyses confirmed the validity of the proposed model by Henseler et al. [9].

**Table 3:** Discriminant validity.

Fornell–Larcker criterion						
	A	DI	IT	PBC	RC	SN
A	0.724					
DI	0.499	0.716				
IT	0.362	0.158	0.786			
PBC	0.504	0.542	0.292	0.708		
RC	0.399	0.338	0.274	0.460	0.819	
SN	0.446	0.287	0.395	0.424	0.402	0.761

Heterotrait-Monotrait ratio (HTMT)						

A						
DI	0.631					
IT	0.434	0.188				
PBC	0.664	0.686	0.363			
RC	0.476	0.404	0.299	0.556		
SN	0.551	0.351	0.448	0.523	0.453	

## 4 Experimental Design, Materials and Methods

This study employed a survey research design to gather primary data from 400 Muslim individuals in Kuala Lumpur, Pahang, Selangor, Pulau Pinang, and Sabah. The data was collected by using a structured questionnaire. The questionnaire was developed with six constructs and 33 items based on a five-point Likert scale (5: strongly agree, 1: strongly disagree). The list of latent variables is as follows: religious commitment, institutional trust, attitude, subjective norm, perceived behavioral control, and donation intention, based on previous studies [1,2]. This dataset used partial least square (PLS) regression due to its advantages in evaluating complex multivariat research model [10]. The convergent validity (Table 2) and discriminant validity of the Fornell–Larceker criterion and Heterotrait-Monotrait ratio (Table 3) were employed on the dataset.

Table 4 summarizes the characteristics of demographics. Specifically, it depicts the socioeconomic background of the respondents in terms of gender, age, education level, household income, marital status, number of dependents, and employment. Of the 400 respondents, the majority (55.8 %) of the respondents are male, while 134 (44.3 %) are female. The four age categories used in this study are 21–30, 31–4, 41–50, and 51 years old and above age group. As noted in Table 1, 126 (31.5 %) of the respondents are aged between 21 and 30 years old, 155 of the respondents (38.8 %) are aged between 31-40 years old; 76, or 19 per cent of the respondents, are in the age group between 41 and 50 years old; and the rest of the respondents (43, or 10.8 %) are aged between 51 years old and above. As per education level, the majority of the respondents (206; 51.5 %) have completed a bachelor's degree, followed by 84 or 21.0 per cent of respondents with a master's degree and above. The remaining respondents include those who have a Malaysian Certificate of Education or SPM with 26 or 6.5 per cent and a Malaysian Higher School Certificate with 53 or 13.3 per cent. This study observed 31 or 7.8 of the respondents obtained a diploma level in their education (Table 5).

**Table 4:** Summary of the PLS-SEM regression.

Path	*β*	*t*-statistics	*p*-value	Decision
Direct effects				
Religious commitment–> Attitude	0.325	6.358	0.000	Accept
Religious commitment–> Subjective norm	0.318	7.589	0.000	Accept
Religious commitment–> PBC	0.411	7.492	0.000	Accept
Institutional trust–> Attitude	0.273	4.730	0.000	Accept
Institutional trust–> Subjective norm	0.308	5.916	0.000	Accept
Institutional trust–> PBC	0.179	2.790	0.005	Accept
Attitude–> Donation intention	0.308	5.401	0.000	Accept
Subjective norm–> Donation intention	-0.018	0.344	0.731	Reject
PBC –> Donation intention	0.394	6.479	0.000	Accept

Indirect effects				

Religious commitment–> Attitude–> Donation intention	0.100	3.837	0.000	Accept
Religious commitment–> Subjective norm–> Donation intention	-0.006	0.342	0.732	Reject
Religious commitment–> PBC–> Donation intention	0.162	4.466	0.000	Accept
Institutional trust–> Attitude–> Donation intention	0.084	3.470	0.001	Accept
Institutional trust–> Subjective norm –> Donation intention	-0.005	0.336	0.737	Reject
Institutional trust–> PBC –> Donation intention	0.071	2.589	0.010	Accept

**Table 5:** Profile of respondents (*N* = 400).

Respondent's profile	*N*	%
Gender (Head of household)		
Male	223	55.8
Female	177	44.3
Age		
21–30	126	31.5
31–40	155	38.8
41–50	76	19.0
51 and above	43	10.8
Education level		
Malaysian Certificate of Education (SPM)	26	6.5
Malaysian Higher School Certificate (STPM)/ Skills Certificate	53	13.3
Diploma	31	7.8
Bachelor Degree	206	51.5
Master Degree and above	84	21.0
Household Income		
B40 (MYR 2500-MYR 4849)	281	70.3
M40 (MYR 4850-MYR 10,959)	99	24.9
T20 (MYR 10,960 and above)	20	4.8
Marital status		
Single	183	45.8
Married	211	52.8
Widow/ widowers	6	1.4
Dependents		
No dependent	133	33.3
1–2 dependents	105	26.3
3–4 dependents	89	22.3
5–6 dependents	58	14.5
7 dependents and above	15	3.8
Employment		
Government/ Private	287	71.8
Self-employed/ Business	81	20.3
Unemployed/ Retired	32	8.0

In terms of household income, the majority of the sample consisted of individuals in low-income groups with 281 or 70.3 per cent and middle-income groups with 99, or 24.9 per cent. The remaining 20 or 4.8 per cent are Muslims, who are classified as high-income groups. As for marital status, 52.8 per cent (211) are married and 45.8 percent (183) are single. The survey data indicated that widows and widowers comprised 1.4 per cent (6) of the study. Observably, the majority of the respondents have dependents, i.e., one and two (26.3 %), three and four (22.3 %), five and six (14.5 %), and seven dependents and above (3.8 %). While 33.3 percent of them do not have any dependents. Furthermore, the majority of the sample is currently working in the government and private sectors (71.8 %), and 20.3 percent of them are self-employed or running some kind of business, while 8.0 percent are unemployed and retired. This study shows a trend toward increased charitable contributions among low-income participants compared to their higher-income counterparts. This finding is consistent with prior research [2], indicating that individuals facing economic challenges often demonstrate heightened levels of generosity towards their communities.

The data set aimed to capture a sample of Muslims, a Malaysian majority group, that are relevant to understanding behavioral intention in charity-giving behavior. While our findings provide valuable insights into the specific aspect we studied, we acknowledge that the sample may not fully represent the diversity of the broader population or societal context. Therefore, external factors such as geographic scope and demographic homogeneity could impact the broader applicability of our results beyond our study sample.

## Limitations

Not applicable.

## Ethics Statements

At the time the study was undertaken, there was no requirement for researchers to obtain ethical approval from local authorities or institutions. Nonetheless, the authors have considered all ethical concerns during the data collection process. This study was undertaken in accordance with the ethical standards and principles in the Helsinki Declaration of 1964. The data gathering process received the verbal consent of the participant prior to the interview session. The participants were informed that the data set was conducted for academic research purposes. Furthermore, their participation is voluntary and confidential, and they can withdraw from the investigation at any time without having to explain why or face a penalty. All personal data obtained from the survey would be kept confidential and analyzed anonymously.

## CRediT authorship contribution statement

**Mohamad Syahmi Mat Daud:** Writing – original draft, Writing – review & editing. **Hairunnizam Wahid:** Supervision, Methodology. **Riayati Ahmad:** Conceptualization, Visualization, Investigation. **Raudha Md. Ramli:** Software, Formal analysis, Data curation.

## Data Availability

Survey Dataset of Factors Affecting Islamic Donation Intention in Malaysia (Original data) (Mendeley Data).
